# Bacterial Extracellular Vesicles and Antimicrobial Peptides: A Synergistic Approach to Overcome Antimicrobial Resistance

**DOI:** 10.3390/antibiotics14040414

**Published:** 2025-04-19

**Authors:** Corina Ciobanasu

**Affiliations:** Department of Exact and Natural Sciences, Institute of Interdisciplinary Research, Alexandru I. Cuza University, Bulevardul Carol I, Nr. 11, 700506 Iasi, Romania; vasilica.ciobanasu@uaic.ro

**Keywords:** AMP-based therapeutics, antimicrobial peptides, antimicrobial resistance, bacterial extracellular vesicles, combinatory therapy, multidrug-resistant pathogens

## Abstract

Antimicrobial resistance is already a major global health threat, contributing to nearly 5 million deaths annually. The rise of multidrug-resistant pathogens has made many infections increasingly difficult to treat. This growing threat has driven the search for alternative therapeutic approaches. Among the most promising candidates are bacterial extracellular vesicles (BEVs) and antimicrobial peptides (AMPs), which offer unique mechanisms of action, potential synergistic effects, and the ability to bypass conventional resistance pathways. This review summarizes the current research on synergistic effects of BEVs and AMPs to overcome antimicrobial resistance.

## 1. Introduction

Antimicrobial resistance represents one of the most pressing global health challenges, significantly limiting the efficacy of conventional treatments for bacterial infections. The overuse and misuse of antibiotics in healthcare, agriculture, and animal husbandry have accelerated the development of resistance, allowing bacteria to evolve defense mechanisms against multiple drug classes [1].

Antimicrobial resistance (AMR) occurs when microorganisms, including bacteria, fungi, viruses, and parasites, evolve mechanisms to withstand the effects of antimicrobial agents. In general, AMR in bacteria is generally classified into two main types: inherent (intrinsic) resistance and acquired resistance [2,3]. Intrinsic resistance refers to the natural, pre-existing ability of certain bacterial species to resist the effects of specific antibiotics without prior exposure or genetic changes. This type of resistance is a fundamental property of the bacterial species and is usually due to structural or functional characteristics that prevent antibiotics from working effectively [4]. Acquired resistance occurs when previously susceptible bacteria develop resistance due to genetic changes. This type of resistance arises through mutations or horizontal gene transfer (HGT) from other resistant bacteria [1].

The rise of multidrug-resistant (MDR) and extensively drug-resistant (XDR) pathogens, such as Methicillin-resistant *Staphylococcus aureus* (MRSA), Carbapenem-resistant *Enterobacteriaceae* (CRE), and Vancomycin-resistant *Enterococci* (VRE), has rendered many infections nearly untreatable. The emergence of such multidrug-resistant pathogens has necessitated alternative therapeutic strategies. Among these, bacterial extracellular vesicles (BEVs) and antimicrobial peptides (AMPs) have emerged as promising candidates due to their unique mechanisms of action, potential for synergy, and ability to circumvent traditional resistance pathways. This review discusses relevant biological aspects of BEVs including biogenesis mechanisms, composition, methods of extraction and purification, and their role in antibiotic resistance. Also, AMPs are described as solutions to overcome conventional resistance mechanisms, especially in combination with BEVs. This synergistic activity is an innovative, still-developing topic but it has a high potential in regard to the antimicrobial resistance problem.

### Mechanisms of Antimicrobial Resistance

**Figure 1:**
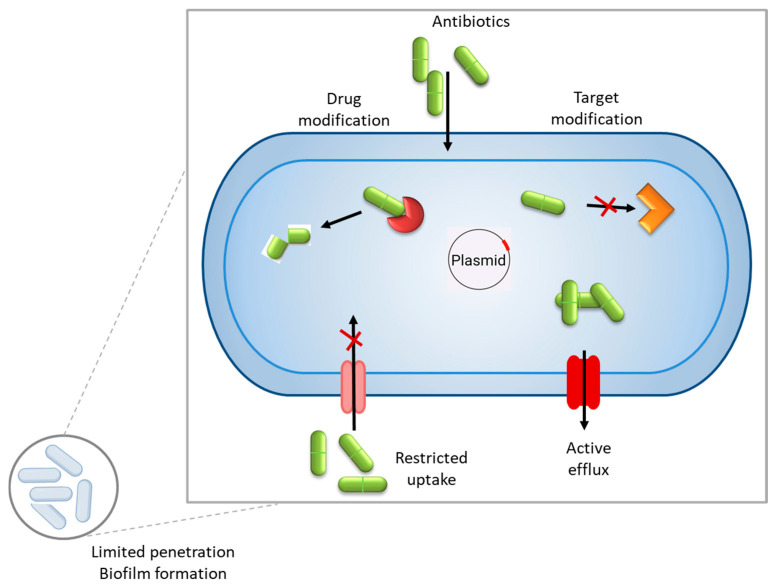
General mechanisms of antibiotic resistance in bacteria include inactivation of the drug, modifying the drug target, limiting the uptake of a drug, and active drug efflux. Within a biofilm, bacterial cells are embedded in a self-produced extracellular polymeric substance (EPS) matrix that acts as a physical and chemical barrier, impeding the penetration of antimicrobial agents.

In general, antimicrobial resistance mechanisms may be divided into four main categories: limiting the uptake of a drug, the inactivation of the drug, the modification of the drug target, and active drug efflux (see Figure 1).

**Enzymatic degradation** or modification of antimicrobial drugs is one of the most common and effective resistance mechanisms by which bacteria produce specific enzymes that degrade or modify antibiotics, rendering them ineffective. β-Lactam antibiotics (e.g., penicillins, cephalosporins, and carbapenems) target bacterial cell wall synthesis. Some bacteria produce β-lactamase enzymes that hydrolyze the β-lactam ring, inactivating the antibiotic [5]. Aminoglycosides (e.g., gentamicin and tobramycin) inhibit bacterial protein synthesis by binding to the 30S ribosomal subunit. Bacteria can produce aminoglycoside-modifying enzymes (AMEs) that modify aminoglycosides via acetylation, phosphorylation, or adenylation, preventing drug binding [6]. Also, some bacteria produce chloramphenicol acetyltransferase (CAT), which inactivates chloramphenicol through acetylation, blocking its ability to bind the ribosome [7]. Macrolide resistance can occur due to esterases or phosphotransferases that modify drugs like erythromycin [8]. Rifamycin resistance is often mediated by ADP-ribosyltransferases, which modify the drug’s structure [9]. Some bacteria evade antibiotics through the alteration of their target sites, preventing drug binding while maintaining essential cellular functions. Macrolides, tetracyclines, aminoglycosides, and oxazolidinones (e.g., linezolid) target ribosomal subunits (30S or 50S). Some bacteria, like *Streptococcus pneumoniae*, can mutate ribosomal proteins or methylate RNA to prevent antibiotic binding [10].

**Biofilms** are a survival strategy of bacteria that enhances survival against antibiotics and host defenses, leading to antibiotic resistance. Bacteria develop biofilms through a complex multistep process that allows them to form structured, resilient communities. The process begins when free-floating (planktonic) bacteria encounter a surface. This initial attachment is reversible and weak, driven by physical forces like van der Waals interactions, hydrophobic effects, or electrostatic charges. Bacteria use appendages such as flagella or pili to approach the surface [11]. Once in contact, specific adhesins (proteins on the bacterial surface) may bind to the substrate, making the attachment more secure and irreversible. Environmental factors, like nutrient availability or surface conditioning (e.g., a layer of organic molecules), can influence this step. After attaching, bacteria begin to proliferate and form microcolonies. They divide and produce extracellular polymeric substances (EPSs), a sticky matrix of polysaccharides, proteins, and DNA that anchors them to the surface and to each other. This EPS layer not only provides structural support but also traps nutrients and protects the growing community from external threats like antibiotics or immune responses. As the biofilm grows, it develops into a complex, three-dimensional structure. This stage involves the formation of water channels within the EPS matrix, which facilitate nutrient and oxygen distribution while removing waste. Genetic regulation fine-tunes the community, with some cells differentiating into distinct roles (e.g., persister cells that resist stress). At this point, the biofilm becomes highly resistant to antibiotics, up to 1000 times more so than planktonic cells, due to the protective EPS and slower metabolic rates of deeper layers [12]. Eventually, some bacteria leave the biofilm to colonize new areas.

Numerous bacterial species possess **efflux pumps**, which are specialized membrane proteins that actively expel a wide range of antimicrobial agents from the cell, significantly contributing to drug resistance. These pumps reduce the intracellular concentration of antibiotics, causing them to be less effective and allowing bacteria to survive in hostile environments. For example, *Enterobacteriaceae*, a family of Gram-negative bacteria commonly found in clinical settings, frequently exhibits the presence of efflux pumps. Notable members, such as *Escherichia coli* and *Klebsiella pneumoniae*, utilize these systems to extrude multiple classes of drugs, including β-lactams, fluoroquinolones, and tetracyclines, thereby enhancing their multidrug resistance profiles [13]. The expression and activity of efflux pumps in *Enterobacteriaceae* are often regulated by complex genetic mechanisms, making them a critical target for overcoming resistance in therapeutic strategies [14].

## 2. Bacterial Extracellular Vesicles

Bacterial extracellular vesicles (BEVs) are membrane-lipid bilayer structures released by bacteria into their surrounding environment. These vesicles are typically spherical and range in size from about 20 to 400 nanometers in diameter, depending on the bacterial species [15]. BEVs are produced by both Gram-positive and Gram-negative bacteria and play diverse roles in bacterial physiology, communication, and interaction with host organisms.

The composition of bacterial extracellular vesicles (BEVs) is highly dynamic and varies significantly based on multiple factors, including the bacterial species, the environmental conditions in which they are produced, and their biological function. Different bacterial species secrete BEVs with distinct molecular compositions, including variations in proteins, lipids, nucleic acids, and metabolites, reflecting their unique genetic and metabolic characteristics [16,17]. Environmental factors such as nutrient availability, stress conditions, pH, and temperature further influence BEV composition, leading to adaptations that optimize bacterial survival and communication. Additionally, the functional role of BEVs, whether in pathogenesis, intercellular communication, antibiotic resistance, or host immune modulation, dictates the selective packaging of specific bioactive molecules [18,19].

**Figure 2:**
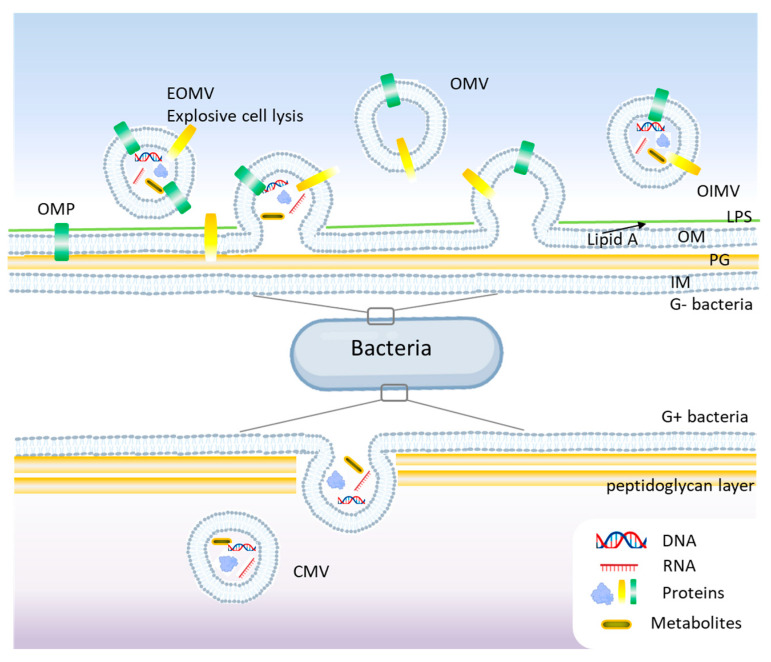
Biogenesis mechanisms of bacterial extracellular vesicles (BEVs) models. In Gram-negative bacteria, blebbing and explosive cell lysis are among the main mechanisms for BEVs, while budding is common in Gram-positive bacteria. OMV, outer membrane vesicle; OIMV, outer-inner membrane vesicle; EOMV, explosive outer membrane vesicle; OM, outer membrane; IM, inner membrane; Lipid A, LPS, lipopolysaccharides; PG, peptidoglycan; OMP, outer membrane proteins.

Gram-negative bacteria possess an outer membrane (OM), a periplasmic space, and an inner membrane (IM), which influences the mechanisms of BEV formation. They primarily release two types of vesicles: outer membrane vesicles (OMVs) and outer-inner membrane vesicles (OIMVs) [20]. OMVs are derived solely from the outer membrane and contain lipopolysaccharides (LPS), outer membrane proteins, phospholipids, and periplasmic components [21]. OIMVs contain both outer and inner membrane components, allowing the packaging of cytoplasmic proteins and nucleic acids [22]. See Figure 2 for a schematic representation of BEVs’ biogenesis.

Unlike Gram-negative bacteria, Gram-positive bacteria lack an outer membrane and have a thick peptidoglycan (PG) layer, which was initially thought to prevent vesicle formation. The formation of membrane vesicles in Gram-positive bacteria is primarily initiated by the expansion of lipid-rich domains within the cytoplasmic membrane [19]. This expansion, combined with the activity of endolysins that degrade the PG, compromises the integrity of the thick peptidoglycan layer, facilitating the release of these vesicles (Figure 2). However, studies showed that Gram-positive bacteria produce BEVs, albeit via different mechanisms [23]. Membrane vesicles (MVs) and cytoplasmic membrane vesicles (CMVs) are found in both Gram-positive and Gram-negative bacteria and are derived from cytoplasmic or plasma membranes. These vesicles carry bacterial proteins, peptidoglycan fragments, and toxins. Some bacteria, like *Pseudomonas aeruginosa* produce tubular or explosive vesicles (explosive outer membrane vesicles, EOMVs) as a defensive or communication strategy [24]. Currently, different mechanisms are reported to understand the generation of BEVs like the blebbing or budding of the cell membrane (CM), explosive cell lysis or bubbling cell death, and the formation of nanotubes [25,26]. Also, the membrane differences between Gram-positive and Gram-negative bacteria impact the biological functions, immunogenicity, and physiological roles of bacterial extracellular vesicles [23]. Since LPS is profoundly immunogenic, its absence in Gram-positive membranes represents a crucial distinction, affecting how bacterial vesicles interact with the host immune system and their potential biomedical applications.

Bacterial extracellular vesicles are highly diverse, with different roles in bacterial communication, pathogenesis, and antibiotic resistance and vary depending on the bacterial strain, environment, and function. Table 1 summarizes the composition, role in antibiotic resistance, and pathogenicity of BEVs from different bacterial strains.

### 2.1. Role of BEVs in Antibiotic Resistance

BEVs play a multifaceted role in promoting antibiotic resistance among bacterial populations through several mechanisms. Firstly, BEVs serve as vehicles for horizontal gene transfer, encapsulating and disseminating resistance genes between bacterial cells, even across species. This transfer enhances the spread of resistance traits within microbial communities, amplifying the resilience of populations against antibiotics. For example, studies have shown that BEVs from *Escherichia coli* and *Pseudomonas aeruginosa* can transfer β-lactam resistance genes to recipient cells, enhancing their survival in the presence of antibiotics like ampicillin or imipenem [38,39]. The degradation or sequestration of antibiotics is another way for BEVs to induce antibiotic resistance. BEVs can contain enzymes, such as β-lactamases, that degrade antibiotics extracellularly before they reach the bacterial cell. For instance, BEVs from methicillin-resistant *Staphylococcus aureus* (MRSA) have been shown to degrade β-lactam antibiotics when exposed to sub-lethal concentrations of ampicillin. Alternatively, BEVs can act as traps, binding antibiotics (e.g., membrane-targeting peptides like colistin) and reducing the effective concentration that reaches the bacterial cell [40]. Also, antibiotic exposure often increases BEV production. Research on MRSA has demonstrated a 22.4-fold increase in BEV secretion when stressed with sub-minimum inhibitory concentrations (MICs) of ampicillin. These vesicles can protect susceptible bacteria in a population by neutralizing antibiotics or exporting harmful substances, like misfolded proteins or excess drugs, out of the cell [41]. Thirdly, BEVs contribute to biofilm formation, a key resistance strategy, by delivering structural components or signaling molecules that fortify the extracellular matrix, making the penetration of antibiotics more difficult. BEVs contribute to biofilm formation by delivering extracellular polymeric substances (e.g., polysaccharides, proteins, and DNA). Biofilms are notoriously resistant to antibiotics due to their physical barrier and altered metabolic state. BEVs from *Pseudomonas aeruginosa*, for example, enhance biofilm growth during the exponential phase, making infections harder to treat [42]. Additionally, BEVs released by resistant bacteria can protect nearby susceptible bacteria. For instance, vesicles from resistant *Klebsiella pneumoniae* have been shown to expel antibiotics via efflux pumps, creating a local environment where susceptible strains can survive longer [43].

### 2.2. Methods for BEV Extraction and Purification

Bacterial extracellular vesicles (BEVs) are valuable tools for various applications, from antimicrobial strategies to vaccine development and gene therapy. To tackle BEVs effectively, it is crucial to isolate them with high purity and yield. BEVs can be extracted using a variety of methods. Differential centrifugation is the most commonly used, while density gradient ultracentrifugation and size-exclusion chromatography (SEC) provide higher purity. The best method depends on the bacterial strain, vesicle properties, and research goals. See Table 2 for a comparison of BEV extraction methods. Differential centrifugation is a commonly used, cost-effective, and an easy to perform method which separates vesicles based on size and density using a stepwise centrifugation process [44]. Density gradient ultracentrifugation uses a sucrose or iodixanol (OptiPrep) gradient to separate vesicles by density [45]. SEC separates vesicles based on size using a gel filtration column [46]. This method may require multiple runs to obtain high yields and is time consuming. Ultrafiltration is a fast method that uses membranes of different pore sizes to retain vesicles while removing small contaminants [47]. Immunoaffinity capture uses antibodies targeting specific BEV markers (e.g., LPS, outer membrane proteins) to pull out vesicles [48]. To achieve both a high yield and high purity, many protocols combine differential centrifugation with density gradient ultracentrifugation or SEC.

## 3. Antimicrobial Peptides

**Figure 3:**
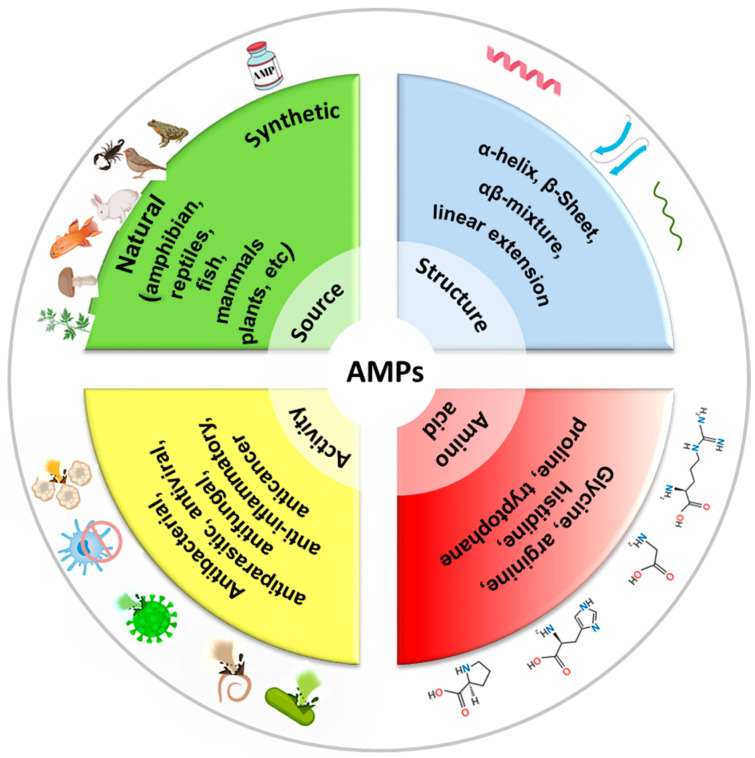
Classification of AMPs by source, structure, activity, and amino acid composition.

Antimicrobial peptides (AMPs) are short chains of amino acids, typically ranging from 10 to 50 residues, which exhibit broad-spectrum antimicrobial activity. They are naturally produced by a wide range of organisms, including humans, animals, plants, and even microorganisms, as part of their innate immune defense systems. AMPs are often cationic (positively charged) and amphipathic (having both hydrophilic and hydrophobic regions), which allows them to interact with and disrupt microbial membranes [3,50]. See Figure 3 for a classification of AMPs. AMPs play a critical role in combating infections, as they have a broad spectrum activity against bacteria (both Gram-positive and Gram-negative), fungi, viruses, and even some parasites [51,52,53]. Also, AMPs kill microbes quickly by physically disrupting their membranes, unlike many traditional antibiotics that target specific metabolic processes. Several models describe how AMPs disrupt membranes, including the barrel-stave, carpet, or toroidal pore models. In the barrel-stave model, AMPs like alamethicin or ceratotoxins insert into the membrane perpendicularly, forming a pore by aligning like staves of a barrel. This allows the leakage of cellular contents, leading to cell death [54]. In the carpet model, AMPs (cecropin for example) coat the membrane surface like a carpet, eventually causing it to disintegrate or form micelles at high concentrations, disrupting membrane integrity [55]. In the toroidal pore model, AMPs like magainin or LL-37 induce the membrane to bend, forming pores lined with both peptides and lipid headgroups, which destabilizes the membrane and causes leakage [56]. Because they attack the microbial membranes rather than specific molecular targets, bacteria have a harder time developing resistance to AMPs compared to conventional antibiotics [57]. Beyond direct antimicrobial effects, some AMPs enhance the immune response by recruiting immune cells or neutralizing inflammatory toxins like lipopolysaccharides (LPSs). In addition to membrane disruption, some AMPs can enter bacterial cells and target critical intracellular components, such as inhibiting DNA and RNA synthesis, blocking protein synthesis and disrupting enzymatic activity [58]. Indolicidin, a peptide derived from the cytoplasmic granules of bovine neutrophils binds bacterial DNA and prevents transcription [59]. Bac7 disrupts ribosome function and inhibits protein synthesis [60]. Other peptides such as protegrins inhibit metabolic enzymes [61]. AMPs can also affect cellular organelles, such as mitochondria, by causing structural damage and finally leading to cell death [62]. More importantly, AMPs can prevent or break down biofilms by interfering with quorum sensing (bacterial communication) or directly degrading the extracellular matrix, making pathogens more vulnerable to immune responses or other treatments [63].

AMPs have emerged as powerful alternatives to traditional antibiotics and due to their unique mechanisms of action and broad-spectrum antimicrobial activity, they also have the ability to overcome conventional resistance mechanisms. These peptides can be used in combination with traditional antibiotics to reduce antibiotic resistance development by preventing bacterial adaptation, and to enhance antibiotic efficacy by making bacteria more susceptible to treatment and lower antibiotic dosages to minimize toxicity and side effects. AMPs are already in use or in development to treat drug-resistant bacterial infections, particularly those caused by MDR pathogens. For example, daptomycin is effective against MRSA and VRE infections [64], polymyxins (colistin, polymyxin B) are last-resort drugs for carbapenem-resistant Gram-negative bacteria [65], and gramicidin is used in topical antibiotics (e.g., eye drops, skin infections) [66]. The combination of colistin and rifampin is more effective than either drug alone against carbapenem-resistant *Klebsiella pneumoniae* [67].

AMPs show potential for the treatment of antibiotic-resistant infections but they still need to be optimized through chemical modifications, nanotechnology-based delivery systems, and bioengineering to enhance their safety, efficacy, and practicality for medical use [19,68]. Table 3 presents some AMPs with challenges and potential solutions.

## 4. Synergistic Effects of AMPs and BEVs Against Antibiotic Resistance

Initially considered cellular debris, BEVs have emerged as highly versatile tools with transformative applications across medicine, biotechnology, vaccine development, drug delivery, and diagnostics [16]. Their natural ability to transport and protect bioactive cargo, transfer information at distal sites, interact with host cells, and modulate biological processes supports their growing significance in scientific research and industry [17]. BEVs play a dual role in medicine, both as mediators of disease and as therapeutic agents. Pathogenic bacteria, such as *Escherichia coli*, *Pseudomonas aeruginosa*, and *Mycobacterium tuberculosis*, release BEVs that deliver virulence factors, contributing to infection and immune evasion [83]. Understanding these mechanisms of delivery provides new solutions into combating bacterial diseases. BEVs have a dual role. For pathogenic bacteria, they serve as delivery vehicles for virulence factors such as toxins, enzymes, or adhesion molecules, that can damage host tissues, manipulate immune responses, or promote infection and antibiotic resistance (detailed at Section 2.1). In bacterial communities, BEVs facilitate communication and resource sharing by transferring genetic material (like antibiotic resistance genes) or signaling molecules between cells. This helps bacteria adapt to stressors, such as antibiotics or nutrient scarcity. For some host-associated bacteria, especially beneficial ones like certain gut microbiota, BEVs can even modulate the immune system in a positive way, promoting tolerance or aiding in gut health [43,84]. Conversely, BEVs from non-pathogenic or engineered bacteria can be utilized for beneficial purposes. For instance, they can stimulate immune responses or deliver therapeutic molecules to target tissues, offering a novel approach to treating infections, inflammation, or even cancer [85]. The combination of BEVs and AMPs represents a relatively new and emerging concept in antimicrobial research. Although both components have been extensively studied individually in the context of bacterial resistance, their combined application is still developing but promising to offer new opportunities for next-generation antimicrobial strategies.

BEVs have the ability to encapsulate and protect the cargo from degradation and function as efficient drug delivery vehicles for bioactive molecules. Their small size enables penetration into tissues and cells, while their natural origin offers biocompatibility advantages over synthetic nanoparticles. BEVs are being studied for delivering antibiotics, anticancer drugs, or RNA-based therapeutics [86,87]. It has been shown that BEVs have innate antimicrobial properties and can be used as natural antibiotics. In a study of the lytic ability of OMVs of Gram-negative bacteria against 17 strains of Gram-negative and gram-pozitive bacteria, it has been shown that OMVs contain PG hydrolases and can destroy other bacteria [88]. In another study, OMVs from *Cystobacter velatus* Cbv34 were found to inhibit the growth of *E. coli* similarly to the antibiotic gentamicin, with low acute inflammatory potential and this underlined the potential for the treatment of these bacterial infections [89]. Another way for OMVs to exert their antimicrobial activity is to induce the production of antimicrobial peptides. For example, OMVs from Nontypeable *Haemophilus influenzae* (NTHI) initiated the release of immunomodulatory cytokine interleukin-8 (IL-8) and also the production of antimicrobial peptide LL-37 by host epithelial cells [90]. Similarly, BEVs from Gram-negative mucosal pathogens *Helicobacter pylori* were shown to induce the expression of human β-defensins HBD2 and HBD3 in epithelial cells [91] and BEVs from *Campylobacter jejuni* and nontypeable *H. influenza* induced the expression of the antimicrobial peptides HBD3 [92].

The distinctive ability of BEVs to transport molecules to the outer membrane of Gram-negative bacteria suggests that they could serve as natural antibiotic carriers, potentially overcoming the challenges associated with delivering antibiotics to these resistant pathogens. Various loading techniques, including electroporation, high-pressure treatment, and sonication, have been explored as effective methods for incorporating diverse therapeutic cargo into BEVs. These techniques enhance the potential of BEVs as drug delivery systems, particularly in improving the encapsulation efficiency of antibiotics [93,94]. A recent study explored extracellular vesicles from lipopolysaccharide-induced hepatocellular carcinoma (HepG2) cells (iEVs) coated with a cationic AMP to combat bacterial sepsis. This approach demonstrated the feasibility of surface-coating EVs with AMPs, improving antibacterial activity (e.g., a 2-fold reduction in minimum bactericidal concentration) against pathogens like *Escherichia coli* and *Staphylococcus aureus* [95]. In another study, antibiotic-loaded BEVs exhibited remarkable stability and biocompatibility, making them highly effective as drug delivery vehicles. Their dual functionality enables them to directly invade bacterial cells, enhancing drug penetration, and also release antibiotics in a controlled manner, maximizing their bactericidal effects. Additionally, when administered orally, these vesicles can protect against intestinal bacterial infections, offering a promising strategy for targeted antibiotic therapy while minimizing systemic side effects [96].

Among the various strategies aimed at reducing antimicrobial use, certain interventions can help prevent the development of multidrug resistance. These include enhanced personal and environmental hygiene, improved management and husbandry practices, vaccination, and emerging technologies such as phage therapy and monoclonal antibody therapy. Of these options, vaccination stands out as one of the most effective approaches to decreasing the reliance on antimicrobial treatments. Surface plasmon resonance (SPR) studies using BEVs from *Klebsiella pneumoniae*, *Acinetobacter baumannii*, and *Pseudomonas aeruginosa* have probed interactions with AMPs, showing that BEVs can serve as realistic models of bacterial membranes. These experiments, while not directly loading AMPs into BEVs, indicate that AMPs can bind to or interact with BEV surfaces, supporting the idea of functionalizing BEVs with AMPs as cargo or coatings [97].

One application of BEVs has been their use in combating *Neisseria meningitidis*, the pathogen that causes meningococcal disease. Meningococcal outbreaks, particularly those involving *Neisseria meningitidis* serogroup B, posed significant public health threats, especially in closed populations such as university campuses and military barracks. In these situations, BEV-based vaccines emerged as a promising solution. These vaccines harness the bacterial vesicles, which contain key antigens that stimulate the immune system to mount a defense against the pathogen, and three BEV-based vaccines, MenBvac, MeNZB, and VA-MENGOCOC-BC, were developed and successfully used in response to meningococcal outbreaks [98]. BEVs can also be used as a delivery vehicle for antigens and adjuvants, facilitating the direct delivery of these components to host cells. In models of H1N1 influenza infection, BEV-based vaccines have been shown to effectively stimulate immune responses, providing protection against the virus. The use of BEVs as a delivery platform for influenza antigens ensures that the immune system can recognize and combat the virus, potentially offering an alternative to traditional vaccine platforms [99]. BEV-based vaccines targeting *A. baumannii* and *S. pneumonia* have shown promise in preclinical models, demonstrating their ability to induce immune protection against these challenging pathogens [100]. OMVs, a subset of BEVs, have been successfully used in vaccines like Bexsero against *Neisseria meningitides* [101,102].

The ability of BEVs to deliver AMPs efficiently to biofilms holds great promise for the treatment of biofilm-associated infections, which are notoriously difficult to manage with traditional antibiotics. BEVs possess membrane-disrupting properties due to their lipid and protein composition, which can weaken biofilm integrity. When combined with AMPs, BEVs enhance the ability to break down the biofilm matrix, making bacterial cells more susceptible to antimicrobial attack [86,103,104]. AMPs like LL-37 and hepcidin have been studied for their ability to disrupt biofilms. Loading these into BEVs could amplify their delivery to bacterial communities [105]. The concept of loading AMPs like LL-37 into BEVs to enhance delivery to bacterial communities is a promising avenue for research. While direct studies on this combination are limited, the individual properties of AMPs and BEVs suggest potential synergistic effects in targeting biofilms and persistent infections.

The combination of BEVs and AMPs represents an exciting avenue for next-generation antibacterial therapies, particularly against antibiotic-resistant bacteria. Their synergistic effects, including enhanced stability, targeted delivery, and biofilm penetration, offer a novel strategy to improve antimicrobial efficacy. Table 4 summarize these synergistic effects of AMPs and BEVs.

## 5. Conclusions and Outlook

Antibiotic resistance is a critical global health threat that severely reduces the effectiveness of standard treatments for bacterial infections. The widespread overuse and improper use of antibiotics in healthcare, agriculture, and animal husbandry have driven the rapid emergence of resistant bacteria, enabling them to develop defense mechanisms against multiple classes of drugs.

Bacterial extracellular vesicles are lipid bilayer structures released by bacteria into their environment. They have different functional roles, ranging from pathogenesis and intercellular communication to antibiotic resistance and host immune modulation. While BEVs contribute to resistance, they also hold promise as tools to fight it. Antimicrobial peptides are short amino acid chains, naturally produced by humans, animals, plants, and microorganisms as part of their innate immune defense. AMPs have emerged as promising alternatives to conventional antibiotics. Their unique mechanisms of action not only enable them to combat a wide range of pathogens but also help bypass traditional resistance mechanisms. Recent research suggests that BEVs and AMPs can synergistically enhance antibacterial efficacy. BEVs can serve as delivery vehicles for AMPs, increasing their stability, bioavailability, and targeted delivery to bacterial cells. Additionally, BEVs may modulate bacterial membrane integrity, making bacteria more susceptible to AMPs. This synergy presents a promising strategy to combat antibiotic-resistant pathogens, but the field is still developing. The studies suggesting BEVs can serve as delivery platforms for AMPs or enhance AMP activity are not so numerous but there is strong theoretical support and early-stage experimental evidence that combining AMPs with vesicle-mediated delivery could enhance antimicrobial effects and potentially bypass antibiotic resistance.

## Figures and Tables

**Table 1:** Composition, resistance, and pathogenicity of bacterial extracellular vesicles (BEVs) by strain.

Bacterial Strain	Type of BEV	Composition	Role in Antibiotic Resistance	Pathogenicity Role	Ref.
*Escherichia coli*	OMVs	Lipopolysaccharides (LPS), heat-labile toxin, heat-stable toxin, virulence factors, small RNAs	Carries β-lactamase, aiding resistance to β-lactam antibiotics	Promotes intestinal infections, urinary tract infections (UTIs)	[27]
*Pseudomonas* *aeruginosa*	OMVs, Explosive Vesicles	Quorum-sensing molecules, phospholipids, alkaline protease, elastase, efflux pump proteins	Carries enzymes that degrade antibiotics, enhances biofilm formation	Facilitates lung infections (CF patients), immune evasion	[19,28]
*Staphylococcus* *aureus*	CMVs	α-hemolysin, peptidoglycan, toxins, adhesins	Transfers methicillin-resistant genes (MRSA), promotes biofilm integrity	Causes skin infections, pneumonia, sepsis	[29]
*Neisseria* *meningitidis*	OMVs	Lipooligosaccharides (LOS), outer membrane proteins, adhesins	Helps evade immune system, limited role in antibiotic resistance	Causes meningitis, septicemia	[30]
*Helicobacter pylori*	OMVs	CagA, VacA toxin, LPS, adhesins	Alters host immune response but limited antibiotic resistance	Promotes gastric ulcers and gastric cancer	[31,32]
*Acinetobacter* *baumannii*	OMVs	OmpA, phospholipids, proteases, outer membrane proteins	Transfers resistance genes (carbapenemase, aminoglycoside resistance)	Causes multidrug-resistant (MDR) infections in hospitals	[33]
*Mycobacterium* *tuberculosis*	MVs	Mycolic acids, lipoproteins, glycolipids, DNA	Protects against antibiotics, modulates host immune response	Enhances survival in host macrophages	[34]
*Vibrio cholerae*	OMVs	Cholera toxin, outer membrane vesicle proteins, quorum-sensing molecules	Helps bacteria resist phage attacks but not majorly involved in antibiotic resistance	Contributes to cholera toxin delivery and infection spread	[35]
*Klebsiella* *pneumoniae*	OMVs	Capsular polysaccharides, lipoproteins, LPS	Facilitates β-lactam resistance, carries carbapenemase	Major cause of nosocomial infections, pneumonia, and sepsis	[36]
*Bacillus subtilis*	MVs	Peptidoglycan, proteins, signaling molecules	Provides defense against antibiotics in soil environments	Beneficial for plant growth and biocontrol rather than pathogenic	[37]

**Table 2:** Comparison of BEV isolation methods.

Method	Purity	Yield	Time Required	Cost	Best Used For	Ref
Differential Centrifugation	Medium, May contain contaminants like protein aggregates and cell debris	Medium	Medium	Medium, requires minimal equipment and reagents.	For initial screening of BEVs, where purity is not the primary concern	[44,49]
Density Gradient Ultracentrifugation	High, with minimal contamination from other cellular components.	Medium	Long	Medium, specific equipment for preparing and processing density gradients.	High-purity BEVs	[45]
Size-Exclusion Chromatography	High, preserves the integrity of the BEVs and their cargo.	Medium	Long	High	Specific vesicle subtypes based on size	[46]
Ultrafiltration	Medium, difficult to remove soluble protein contaminants	High	Fast	Medium	Large-scale prep	[47]
Immunoaffinity Capture	High	Low	Very Long	High	Specific BEV isolation	[48]

**Table 3:** AMP applications, challenges, and solutions.

AMP	Applications	Challenges	Potential Solutions	Ref.
Colistin (Polymyxin E)	antibiotic for MDR Gram-negative bacteria (*Klebsiella pneumoniae*, *Pseudomonas aeruginosa*, *Acinetobacter baumannii*).	Nephrotoxicity, neurotoxicity, resistance development (mcr-1 gene).	Liposomal formulations, combination therapy with rifampin, polymyxin derivatives with reduced toxicity.	[69,70]
Daptomycin	Effective against MRSA, VRE, and drug-resistant *Enterococcus* species.	Reduced activity in lung surfactants (not effective for pneumonia).	Novel formulations, nanoparticle delivery systems.	[71,72]
LL-37 (Human Cathelicidin)	Prevents biofilm formation (*Pseudomonas aeruginosa*), immunomodulatory, wound healing.	Susceptible to enzymatic degradation in vivo.	Chemical modifications (D-amino acids, cyclization), nanoparticle-based delivery.	[73]
Indolicidin	Inhibits DNA synthesis in *E. coli*, prevents biofilms.	Cytotoxicity at high concentrations.	Peptide engineering for improved selectivity.	[74]
Gramicidin	Used in topical antibiotics (eye and skin infections).	Hemolytic toxicity limits systemic use.	Liposomal encapsulation to reduce toxicity.	[75]
Magainins (Frog-Derived AMPs)	Potential use in antiviral and antifungal therapies, broad antimicrobial spectrum.	Poor stability in the bloodstream.	PEGylation (PEG-modified peptides), hybrid peptides.	[76,77]
Defensins	Found in human neutrophils, effective against Gram-positive and Gram-negative bacteria.	Limited large-scale production.	Recombinant peptide production (synthetic biology).	[78,79]
Bactenecins	Inhibits Gram-negative pathogens, anti-biofilm properties.	Rapid degradation in the bloodstream.	Protease-resistant peptide analogs.	[80]
Protegrins	Used in oral care, shows activity against drug-resistant *Pseudomonas* strains.	Toxicity in mammalian cells.	Sequence modifications to enhance selectivity.	[81]
Histatins	Antifungal AMPs (active against *Candida albicans*), used in oral care products.	Enzymatic degradation in saliva.	Hybrid peptide engineering.	[82]

**Table 4:** The hypothetical synergistic effects of AMPs and BEVs.

Mechanism	Role of BEVs	Role of AMPs	Synergistic Effect
Targeted Drug Delivery	BEVs act as natural nanocarriers, delivering AMPs directly to bacterial cells.	AMPs attack bacterial membranes, disrupting integrity.	Enhanced local AMP concentration and specificity for resistant bacteria.
Membrane Permeabilization	BEVs fuse with bacterial membranes, increasing permeability.	AMPs create pores in membranes, leading to bacterial lysis.	Stronger membrane disruption, causing rapid bacterial death.
Overcoming Efflux Pumps	BEVs bypass efflux pumps, preventing bacteria from expelling AMPs.	AMPs disrupt efflux pump proteins, making bacteria more vulnerable.	Increased retention of AMPs inside bacterial cells.
Biofilm Penetration	BEVs carry biofilm-degrading enzymes or AMPs to bacterial communities.	AMPs break down biofilm structures, increasing bacterial exposure.	Biofilm eradication, making bacteria more susceptible to treatment.
Gene Transfer and Regulation	BEVs carry regulatory RNA/proteins that modulate bacterial gene expression.	AMPs interfere with bacterial gene transcription and translation.	Disruption of resistance gene expression, reducing bacterial survival.
Immunomodulation	BEVs influence host immune responses by modulating inflammation.	AMPs act as immune activators, enhancing pathogen clearance.	Strengthened innate immune defense against infections.
